# The Book World of Medicine and Science

**Published:** 1895-10-05

**Authors:** 


### The Book World of Medicine and Science.

The Volunteer Surgeon's Guide. By Surgeon-Captain R. R. Sleman, "Artists" R.V.

This is a real guide book. No attempt is made to render it light reading, but everything is formally arranged in numbered sections and paragraphs, so that cross references are easy. A careful perusal of the book shows how absolutely necessary it is that volunteer surgeons should possess such a guide. The more completely the volunteer service becomes a part of the general military organisation of the country the more essential it is that they should completely understand the regulations by which they are governed. This applies to medical officers quite as much as to others, especially since the large development of the service during recent years, the growth of the Volunteer Medical Staff Corps, and the formation of Brigade Bearer Companies. We can recommend this book as giving a very complete summary of the many regulations which affect the service, all of which must be understood if efficiency is to be attained.

The Home Doctor: A Household Guide for Use in Illness. By Florence Stacpoole. (Paisley: Alexander Gardner.)

This is a title which it is well not to take too seriously. It is, indeed, of some interest as a specimen of book-making; on the one hand showing how by a careful perusal of medical books an ingenious person can produce a book on medical topics, and on the other giving us some idea of the sort of popular work on medicine which can be produced for threepence.

There is a sweet simplicity in the alphabetical arrangement of diseases which disarms criticism. Under its guidance we glide easily from chilblains to chicken-pox, from fits to flatulence, or from rickets to ringworm, and the classification of diseases is as simple as A, B, C. Unfortunately in practice diagnosis declines to be made correspondingly easy; and while we are free to admit that in the treatment which is recommended for the various diseases mentioned there is a good deal which is quite appropriate, as in fact was to be expected from the way the book has evidently been put together, we are at the same time lost in wonder as to how the good people for whom it is written are to find out what is the matter with them, and which particular remedy they are to apply.

There is a little chapter at the end entitled "Useful Hints" which no doubt will be of service, and there are many suggestions scattered through the pages as to the necessity of sending for the doctor which may with advantage be followed. At the same time, we would suggest that there is sometimes a certain redundancy in the advice proffered. Under the head of Pleurisy, for example, it is very properly stated that "It is a dangerous illness, and must not be home-doctored," and Dr. Clifford Allbutt is quoted as to the importance of early attention. What, then, after this is the use of giving detailed directions as to the treatment, including a quotation from a well-known text-book in regard to the method of securing local rest by the application of adhesive plaster?

The prescriptions, which appear in considerable number, are stated to have been "specially written for these pages by a registered medical practitioner of experience," but we doubt whether that guarantee will make it any the easier to decide in what cases they should be used. To this, however, there are exceptions, and the very numerous class of people who suffer from "pimples on the face" must experience considerable mental peace when they discover that, on condition of suffering at the same time from "indigestion and pains after eating," their course is clear, and that only one prescription is offered to them. Perhaps, however, their hopes of cure will be damped on arriving at the chemist and offering a prescription containing "hydroceanic acid"! Even what one may call the common-sense directions are not always properly expressed. What are we to think of the following: "Sleeping on feather beds and much sitting on soft seats is to be avoided; a cane-bottom chair should be used instead.' Surely a cane-bottomed chair is a poor substitute for a feather bed; yet so says the oracle. This threepenny worth of medical knowledge will well repay a careful perusal—for within it are hidden many gems.

The Twentieth Century Practice of Medicine. (London: Sampson Low, Marston, and Co.)

The dawn of the new century is heralded by the announcement of a great medical work, "The Twentieth Century Practice of Medicine," to be published in twenty volumes by Messrs. Sampson Low, Marston, and Co. It is no small undertaking to attempt to gather together the knowledge at our disposal in all the various branches of medical science, yet so great has been the progress during recent years, and from so completely different a standpoint is disease now regarded, from what is was only a few years ago, that it is necessary that some such effort should be made. The formation of a medical library is indeed becoming an increasingly difficult task; text-books are too brief, while the monographs of original workers are generally either too scattered or too ponderous, and are often inaccessible. "The Twentieth Century Practice of Medicine" proposes to get over this difficulty by inviting leading men of all nations to contribute articles on their own subjects. This, of course, is no new idea, but so far as we can judge from the list of the contributors we doubt whether such a truly international work on medicine has ever before been introduced. It goes without saying that American writers figure largely in the list, but we should add that, considering the immense English-speaking population which now occupies that great continent, and the number of active centres of scientific investigation scattered throughout its length and breadth, America by no means appears unduly prominent in the contents bill, and we would especially add that all Europe seems to have been ransacked for special authorities on subjects of recent investigation. On turning over the list the eye is caught by names well known in England. Fielding Blandford, Radcliffe Crocker, Sir Dyce Duckworth, Sir Joseph Fayrer, Hurry Fenwick, Reginald Harrison, Sir George Humphry, Jonathan Hutchinson, Norman Kerr, George Murray, Ernest Sansom, Sir T. Grainger Stewart, and Dawson Williams are names which give a certain guarantee. We shall doubtless review the contents of the volumes as they appear, but we may state that those which we have already seen are well printed and illustrated, and quite worthy of the firm by whom they are being issued.

